# A Novel Bispecific Antibody Targeting EGFR and VEGFR2 Is Effective against Triple Negative Breast Cancer via Multiple Mechanisms of Action

**DOI:** 10.3390/cancers13051027

**Published:** 2021-03-01

**Authors:** Nishant Mohan, Xiao Luo, Yi Shen, Zachary Olson, Atul Agrawal, Yukinori Endo, David S. Rotstein, Lorraine C. Pelosof, Wen Jin Wu

**Affiliations:** 1 Division of Biotechnology Review and Research 1, Office of Biotechnology Products, Office of Pharmaceutical Quality, Center for Drug Evaluation and Research, U.S. Food and Drug Administration, Silver Spring, MD 20993, USA; Nishant.Mohan@fda.hhs.gov (N.M.); luo.xiao0205@gmail.com (X.L.); Yi.Shen@fda.hhs.gov (Y.S.); zro2@georgetown.edu (Z.O.); Atul.Agrawal2@fda.hhs.gov (A.A.); Yukinori.Endo@fda.hhs.gov (Y.E.); 2 Division of Compliance, Office of Surveillance and Compliance, Center for Veterinary Medicine, U.S. Food and Drug Administration, Derwood, MD 20855, USA; David.Rotstein@fda.hhs.gov; 3 Division of Oncology 3, Office of Oncologic Diseases, Center for Drug Evaluation and Research, U.S. Food and Drug Administration, Silver Spring, MD 20993, USA; Lorraine.Pelosof@fda.hhs.gov

**Keywords:** EGFR, VEGFR2, bispecific antibody (BsAb), triple-negative breast cancer (TNBC), autocrine and paracrine

## Abstract

**Simple Summary:**

Triple-negative breast cancer (TNBC) accounts for approximately 10–20% of all diagnosed breast cancers and is often associated with a poor prognosis. There is therefore an urgent need to develop novel and targeted therapeutic approaches against TNBC. Epidermal growth factor receptor (EGFR) and vascular endothelial growth factor receptor 2 (VEGFR2) are prominent therapeutic protein targets that are frequently overexpressed in TNBC. In this investigation, we developed a novel bispecific antibody (BsAb) targeting EGFR and VEGFR2 (designated as anti-EGFR/VEGFR2 BsAb) and investigate its anti-tumor activity using TNBC cellular and xenograft mouse models. Data from these studies indicate that anti-EGFR/VEGFR2 BsAb elicited more comprehensive anti-tumor activity via multiple mechanisms of action, including direct inhibition of EGFR and VEGFR2 in TNBC cells, and disruption of autocrine and paracrine pathways in TNBC and endothelial cells, compared to the individual parental mAbs. Our data suggest that this novel BsAb warrants further investigation as a targeted antibody therapeutic to treat TNBC.

**Abstract:**

Both EGFR and VEGFR2 frequently overexpress in TNBC and cooperate with each other in autocrine and paracrine manner to enhance tumor growth and angiogenesis. Therapeutic mAbs targeting EGFR (cetuximab) and VEGFR2 (ramucirumab) are approved by FDA for numerous cancer indications, but none of them are approved to treat breast cancers. TNBC cells secrete VEGF-A, which mediates angiogenesis on endothelial cells in a paracrine fashion, as well as promotes cancer cell growth in autocrine manner. To disrupt autocrine/paracrine loop in TNBC models in addition to mediating anti-EGFR tumor growth signaling and anti-VEGFR2 angiogenic pathway, we generated a BsAb co-targeting EGFR and VEGFR2 (designated as anti-EGFR/VEGFR2 BsAb), using publicly available sequences in which cetuximab IgG backbone is connected to the single chain variable fragment (scFv) of ramucirumab via a glycine linker. Physiochemical characterization data shows that anti-EGFR/VEGFR2 BsAb binds to both EGFR and VEGFR2 in a similar binding affinity comparable to parental antibodies. Anti-EGFR/VEGFR2 BsAb demonstrates in vitro and in vivo anti-tumor activity in TNBC models. Mechanistically, anti-EGFR/VEGFR2 BsAb not only directly inhibits both EGFR and VEGFR2 in TNBC cells but also disrupts autocrine mechanism in TNBC xenograft mouse model. Furthermore, anti-EGFR/VEGFR2 BsAb inhibits ligand-induced activation of VEGFR2 and blocks paracrine pathway mediated by VEGF secreted from TNBC cells in endothelial cells. Collectively, our novel findings demonstrate that anti-EGFR/VEGFR2 BsAb inhibits tumor growth via multiple mechanisms of action and warrants further investigation as a targeted antibody therapeutic for the treatment of TNBC.

## 1. Introduction

Bispecific antibodies (BsAbs) have been under study for several decades and a wide variety of BsAb formats and techniques are in development [1]. Theoretically, BsAbs are genetically engineered, recombinant antibodies that can target two antigens simultaneously and have shown promise in the cancer therapeutics area [2,3]. To date, two BsAbs were approved by the U.S. Food and Drug Administration (FDA, https://www.fda.gov/), over 85 are in clinical development and more than 20 different commercialized technology platforms are available for BsAb generation [4]. Selecting the correct molecular format and suitable therapeutic target for BsAbs are important considerations for clinical development. Among the several IgG or IgG-like BsAb formats, the dual variable domain immunoglobulins (DVD-Ig) are a widely popular symmetrical full-length IgG-like platform in which a single chain variable domain (scFv) of one monoclonal antibody (mAb) is tethered to a second mAb via a short peptide linker [5]. DVD-Ig BsAbs molecules are tetravalent, and thus can potential bind to four antigens simultaneously, displaying better efficacy.

Triple negative breast cancer (TNBC), which represents about 15–20% of all breast cancers, remains a significant clinical challenge due to lack of expression of druggable targets such as estrogen receptor, progesterone receptor and HER2 [6,7]. TNBC tumors do however frequently overexpress human epidermal growth factor receptor (EGFR), which can be used as predictor for anti-EGFR targeted therapies [8,9]. EGFR expression in breast cancer is associated with an aggressive phenotype, higher proliferation rate and greater genomic instability [10]. Aberrant EGFR signaling promote tumor cell proliferation, migration and survival thus making it an attractive target for anti-cancer therapy. Several anti-EGFR mAbs (cetuximab, necitumumab, and panitumumab) have been developed and approved for treatment of various indications. However, no EGFR-targeted mAbs are currently approved for the treatment of breast cancer. Although cetuximab inhibited the growth of EGFR-expressing TNBC cell line [11], data from a phase II clinical study showed that cetuximab did not have a favorable clinical outcome in metastatic TNBC patients [12].

The vascular endothelial growth factor (VEGF) family and their receptors (VEGFRs) play a crucial role in tumor progression, metastasis and angiogenesis [13,14]. Microvascular density, a prognostic factor in invasive breast cancer, occur at a significantly higher rate in TNBC and basal-like tumors, compared with non-TNBC and non-basal like tumors [15]. Breast cancer cells secrete VEGF-A, which further activates VEGF receptors on surface of cancer cells indicate presence of autocrine loop that enables the breast cancer cells to promote their own growth and survival [16]. Patients with operable TNBC contain significantly higher levels of VEGFs [17], suggesting that anti-VEGF/VEGFR2-targeted therapies could be exploited for the treatment of TNBC tumors. A number of mAbs targeting VEGF/VEGFR signaling pathway are used in the clinic for treatment of a variety of cancers, but none of them has received approval yet for the treatment of TNBC patients. Ramucirumab, which binds to VEGFR2 and blocks ligand-stimulated activation, did not show a statistically significant clinical outcome in metastatic breast cancer patients [18].

There is an extensive autocrine and paracrine crosstalk between EGFR and VEGFR signaling pathways to promote tumor growth and angiogenesis [19,20]. In autocrine signaling, cells secrete extracellular ligands which bind to the receptors on same cells and initiate signal transduction, whereas, in paracrine signaling, secreted growth factors from one cell target the neighboring cells to initiate signaling cascade. Breast cancer cells produce VEGF-A which further activates Erk and Akt signaling and promotes their own growth and survival by autocrine signaling loop [21]. Moreover, activation of the EGFR pathway increases the production of VEGF-A in tumor cells which interacts with VEGFR2 on endothelial cells and promotes the proliferation, migration and differentiation of endothelial cells in a paracrine manner [19]. Therefore, co-targeting of these two receptors using a BsAb may disrupt the autocrine and paracrine cooperation of EGFR/VEGFR2 signaling and inhibit tumor growth and survival in TNBC. For this purpose, we have generated a novel BsAb directed against EGFR and VEGFR2 (anti-EGFR/VEGFR2) using DVD-Ig approach in which single chain variable fragment (scFv) of ramucirumab was fused to full-length cetuximab IgG via a flexible glycine serine linker. The novel anti-EGFR/VEGFR2 BsAbwas produced and purified using an Exp293 mammalian expression system and its anti-tumor effects were studied across different TNBC cell lines and tumor xenograft model.

## 2. Results

### 2.1. Construction, Production and Purification of BsAb

**Figure 1:**
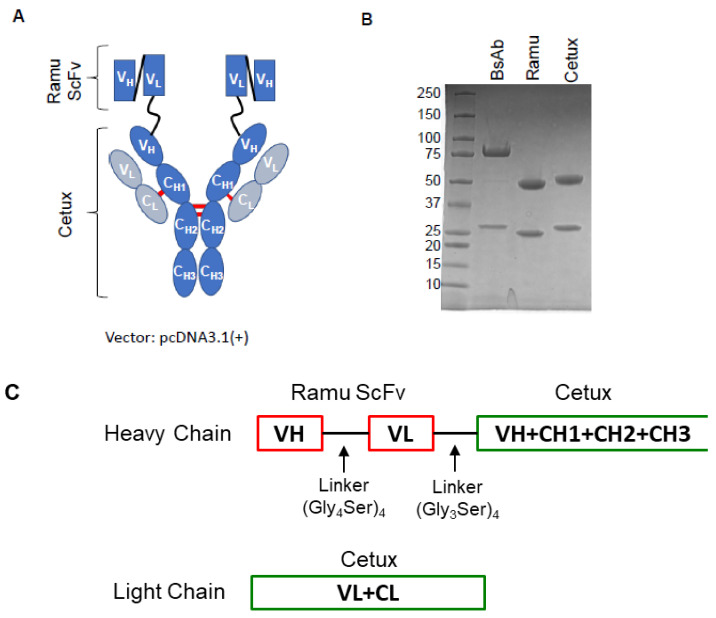
Construction and production of anti-EGFR/VEGFR2 bispecific antibody (BsAb). (**A**) Schematic representation of the structure of anti-EGFR/VEGFR2 BsAb which shows that anti-EGFR/VEGFR2 BsAb is composed of cetuximab IgG linked to the scFv of ramucirumab via a glycine linker. (**B**) SDS-PAGE analysis showing the heavy and light chains of purified anti-EGFR/VEGFR2 BsAb (BsAb), ramucirumab (ramu) and cetuximab (cetux) under reducing conditions. (**C**) Schematic representation of heavy chain and light chain constructs of anti-EGFR/VEGFR2 BsAb showing structural elements along with linker information.

The anti-EGFR/VEGFR2 BsAb is composed of an IgG backbone of EGFR-targeting mAb, cetuximab, and scFv of VEGFR2-targeting ramucirumab connected through a glycine-serine linker (Figure 1A). Generation and production of anti-EGFR/VEGFR2 BsAb was performed by standard DNA recombinant technologies, and BsAb was purified from cell culture supernatant by protein A chromatography and analyzed by SDS-PAGE. As shown in Figure 1B, the purified BsAb yielded a heavy chain protein band of ~80 kDa and light chain protein band of ~25 kDa under reducing conditions of SDS-PAGE. The parental cetuximab (Cetux) and ramucirumab (Ramu) were used as controls which yielded two major bands: IgG heavy chain (~50 kDa) and the IgG light chain (~25 kDa). Figure 1C shows the constructs of heavy chain and light chain of anti-EGFR/VEGFR2 BsAb along with the linker information.

### 2.2. Binding Characterization of Anti-EGFR/VEGFR2 BsAb

**Figure 2:**
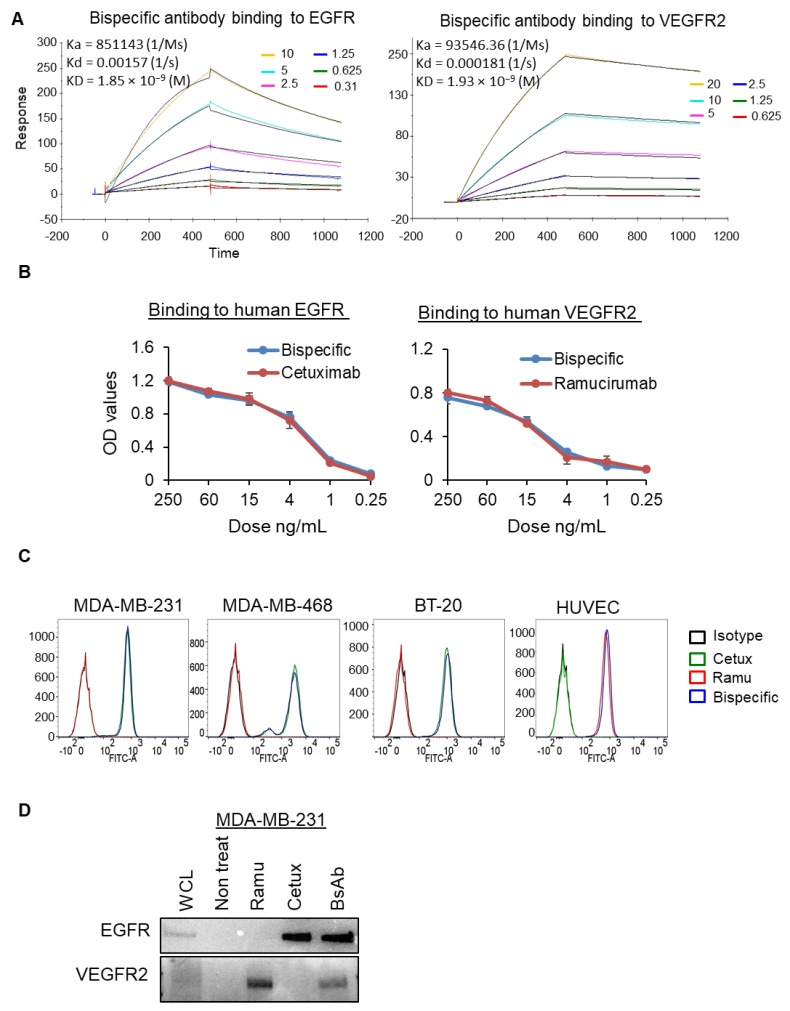
Binding activities of BsAb: (**A**) Surface plasmon resonance sensor grams showing the binding kinetics of anti-EGFR/VEGFR2 BsAb to antigens EGFR and VEGFR2 as detected by a Biacore T200 optical biosensor instrument. Black line represents the constant concentration of anti-EGFR/VEGFR2 BsAb (8 µg/mL) and colored lines represent the nM concentrations of antigens (EGFR or VEGFR2). (**B**) ELISA binding assay showing that anti-EGFR/VEGFR2 BsAb binds to both EGFR and VEGFR2 comparable with cetuximab and ramucirumab, respectively. (**C**) Flow cytometry experiment was performed to assess the binding of anti-EGFR/VEGFR2 BsAb, cetuximab, ramucirumab with cell surface EGFR and VEGFR2 expressed on MDA-MB-231, BT-20, MDA-MB-468 and HUVEC cells. Human IgG was used as isotype control. (**D**) Whole cell lysate of MDA-MB-231 cells was subjected to co-immunoprecipitation assay to assess the binding of anti-EGFR/VEGFR2 BsAb with EGFR and VEGFR2 comparable to parental antibodies. Unprocessed western blot images are available in Figure S4.

The binding affinity of anti-EGFR/VEGFR2 BsAb against each target antigens was determined by a Biacore T200 optical biosensor instrument, enzyme linked immunosorbent assay (ELISA), flow cytometry, and co-immunoprecipitation assay. The binding kinetics data from Biacore indicated that affinity of anti-EGFR/VEGFR2 BsAb to recombinant EGFR and VEGFR2 is similar to that of the cetuximab and ramucirumab, respectively (Figure 2A and Figure S1A,B). The equilibrium dissociation constant (KD) value of anti-EGFR/VEGFR2 BsAb to EGFR was 1.84602 × 10^−9^ and cetuximab to EGFR was 1.18842 × 10^−9^. Similarly, the KD values of anti-EGFR/VEGFR2 BsAb to VEGFR2 was 1.93488 × 10^−9^ and ramucirumab to VEGFR2 was 9.49034 × 10^−10^. Using ELISA method, we determined the dose-dependent binding profiles of the anti-EGFR/VEGFR2 BsAb against immobilized human EGFR and VEGFR2 and compared with cetuximab and ramucirumab respectively (Figure 2B). The ELISA optical density (OD) values affirmed that anti-EGFR/VEGFR2 BsAb bound to the extracellular domains of both EGFR and VEGFR2, which were comparable to the values of each single antigen-targeting antibody, indicating that anti-EGFR/VEGFR2 BsAb maintained in the format of IgG backbone linked with scFv (Figure 2B). Flow cytometric binding assay was performed to confirm the binding ability of anti-EGFR/VEGFR2 BsAb with EGFR and VEGFR2 antigens expressed on the cell surface. Anti-EGFR/VEGFR2 BsAb demonstrated high binding signal in EGFR expressing cells (MDA-MB-231, BT-20 and MDA-MB-468) cells similar to cetuximab and in VEGFR2 expressing cells (HUVEC) similar to ramucirumab (Figure 2C). Table 1 shows quantitative data for binding of anti-EGFR/VEGFR2 BsAb, trastuzumab or ramucirumab to the cell surface. Finally, we performed co-immunoprecipitation assay using MDA-MB-231 cells and data showed that anti-EGFR/VEGFR2 BsAb was co-immunoprecipitated with both EGFR and VEGFR2 (Figure 2D and Figure S4). Taken together, binding data indicate that anti-EGFR/VEGFR2 BsAb binds to both EGFR and VEGFR2 equivalent to parental mAbs.

### 2.3. Anti-EGFR/VEGFR2 BsAb Effectively Inhibits Cellular Proliferation in TNBC Cells

**Figure 3:**
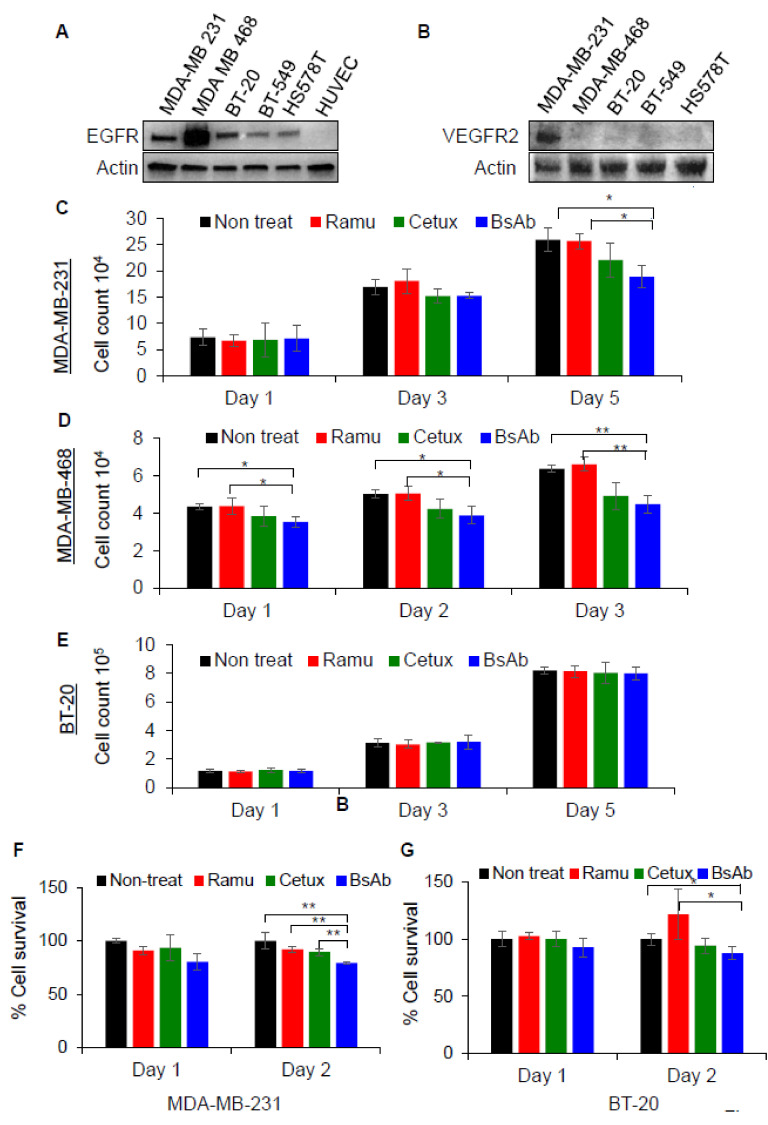
Anti-EGFR/VEGFR2 BsAb inhibits the growth of TNBC cells. (**A**,**B**) The WCL of MDA-MB-231, MDA-MB-468, BT-20, BT-549, HS578 T breast cancer cells and HUVEC cells was subjected to western blot analysis to evaluate the endogenous expression of EGFR and VEGFR2. Actin was used as loading control. (**C**) The inhibition of cell proliferation profile of MDA-MB-231 cells was evaluated by trypan blue exclusion assay after treatment with anti-EGFR/VEGFR2 BsAb, cetuximab, ramucirumab or non-treated cells at day 1, 3 and 5. (**D**) The inhibition of cell proliferation profile of MDA-MB-468 cells after treatment with anti-EGFR/VEGFR2 BsAb, cetuximab, ramucirumab or non-treated cells at day 1, 2 and 3. The cell numbers were directly counted by using Celigo Imaging System. (**E**) BT-20 cells were subjected to trypan blue exclusion assay, and cell numbers were counted at day 1, 3 and 5 after treatment with anti-EGFR/VEGFR2 BsAb, cetuximab, ramucirumab. Cell viability of MDA-MB-231 cells (**F**) and BT-20 cells (**G**) was determined by MTT assay after treatment with cetuximab, ramucirumab, anti-EGFR/VEGFR2 BsAb or non-treated cells at day 1 and day 2. *, *p* < 0.05; **, *p* < 0.01. Unprocessed western blot images are available in Figure S4.

In order to investigate the anti-tumor activity of anti-EGFR/VEGFR2 BsAb in TNBC cell lines, we assessed the levels of EGFR and VEGFR2 expression in a panel of TNBC cells. Expression analysis using western blotting showed that MDA-MB-468 cells had the highest expression of EGFR whereas MDA-MB-231 and BT-20 cell contained slightly lower EGFR (Figure 3A and Figure S4). The highest VEGFR2 expression was detected in MDA-MB-231 cells among all TNBC cells tested (Figure 3B and Figure S4). Based on expression data, MDA-MB-231, BT-20 and MDA-MB-468 cells were subjected to cell proliferation inhibition assay after treatment with cetuximab, ramucirumab and anti-EGFR/VEGFR2 BsAb. Trypan blue cell proliferation inhibition assay data showed that anti-EGFR/VEGFR2 BsAb significantly inhibited the growth of MDA-MB-231 cells on day 5 as compared to the non-treat and ramucirumab treatment (Figure 3C). For the MDA-MB-468 cells, anti-EGFR/VEGFR2 BsAb significantly suppressed the cell proliferation on day 1, 2 and 3 of treatments when compared with non-treat and ramucirumab-treated cells (Figure 3D). No significant differences in the inhibition of cell proliferation were observed between cetuximab and anti-EGFR/VEGFR BsAb in both cell lines (Figure 3C,D). The trypan blue cell proliferation inhibition assay was also performed in MDA-MB-231 and MDA-MB-468 cells to determine whether exogenously added EGF can affect the inhibition of cell proliferation by mAbs and anti-EGFR/VEGFR2 BsAb. As shown in Figure S2A (MDA-MB-231) and Figure S2B (MDA-MB-468), EGF did not promote cell proliferation in both cell lines, and anti-EGFR/VEGFR2 BsAb exhibited similar inhibitory properties as shown in Figure 3C,D. No significant growth inhibition by anti-EGFR/VEGFR2 BsAb was observed in BT-20 cells (Figure 3E). MTT cell viability assay was additionally performed in MDA-MB-231 and BT-20 cells after treating cells with anti-EGFR/VEGFR2 BsAb and mAbs. Data from MDA-MB 231 cells (Figure 3F) were consistent with that shown in Figure 3C. However, in BT-20 cells, significant inhibitory effect of anti-EGFR/VEGFR2 BsAb on cell viability was detected at day 2 treatment as compared to non-treat and ramucirumab treatment (Figure 3G), suggesting that MTT cell viability assay appears to be a more sensitive method to detect growth inhibition in BT-20 cells. The overall cell proliferation data indicate that anti-EGFR/VEGFR2 BsAb can inhibit the cell proliferation of TNBC cells in vitro.

### 2.4. Anti-EGFR/VEGFR2 BsAb Shows Antitumor Activity in Tumor Xenograft Model

**Figure 4:**
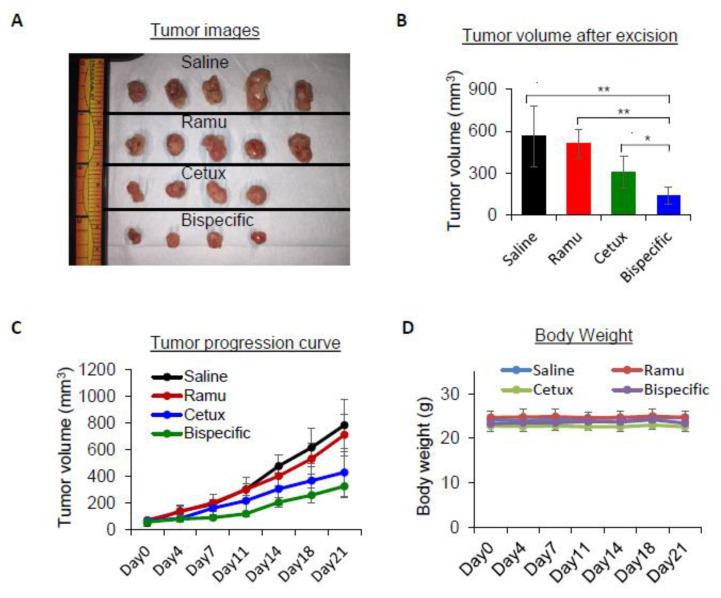
In vivo anti-tumor activity of anti-EGFR/VEGFR2 BsAb in nude mice bearing MDA-MB-231 tumor cells. Treatment condition; saline, ramucirumab, cetuximab, and anti-EGFR/VEGFR2 BsAb given intraperitoneally at 10 mg/kg body weight twice a week. (**A**) The images of the isolated MDA-MB-231 tumors at the end of the treatments. (**B**) The volume of tumors after excising the tumors from mice at the end of treatment course on day 21; *, *p* < 0.05; **, *p* < 0.01. (**C**) The growth of tumors in different groups over the course of treatment. (**D**) Measurement of body weight in different groups over the course of treatment.

To determine whether the anti-proliferative activity of anti-EGFR/VEGFR2 BsAb observed in cellular models can translate to similar activity in vivo, we evaluated the anti-tumor activity in athymic nude mice bearing MDA-MB-231 xenografts. When the tumor volume reached 50–100 mm^3^, mice were intraperitoneally injected with 10 mg/kg cetuximab, ramucirumab, anti-EGFR/VEGFR2 BsAb or saline twice a week. The images of the excised tumor from different treatment groups are shown in Figure 4A. Based on calculation of excised tumor volume, the mice receiving anti-EGFR/VEGFR2 BsAb showed significant inhibition of the tumor growth compared to the saline and individual treated mice (Figure 4B). Notably, treatment with 10 mg/kg of each individual mAb was less effective at inhibiting tumor growth than treatment with anti-EGFR/VEGFR2 BsAb. Tumor progression curve as shown in Figure 4C indicate that anti-EGFR/VEGFR2 BsAb-treated mice had slow tumor growth over the course of treatment compared to other treatment groups (Figure 4C). Furthermore, no significant weight loss was observed in the mice during treatment period, suggesting that cetuximab, ramucirumab or anti-EGFR/VEGFR2 BsAb treatment did not cause any systemic toxicity in tumor-bearing mice (Figure 4D). Taken together, these findings suggest that anti-EGFR/VEGFR2 BsAb has better inhibitory activity than the individual monospecific mAb in in vivo TNBC xenograft mouse model.

### 2.5. Anti-EGFR/VEGFR2 BsAb Inhibits Ligand-Induced EGFR and VEGFR2 Signaling

**Figure 5:**
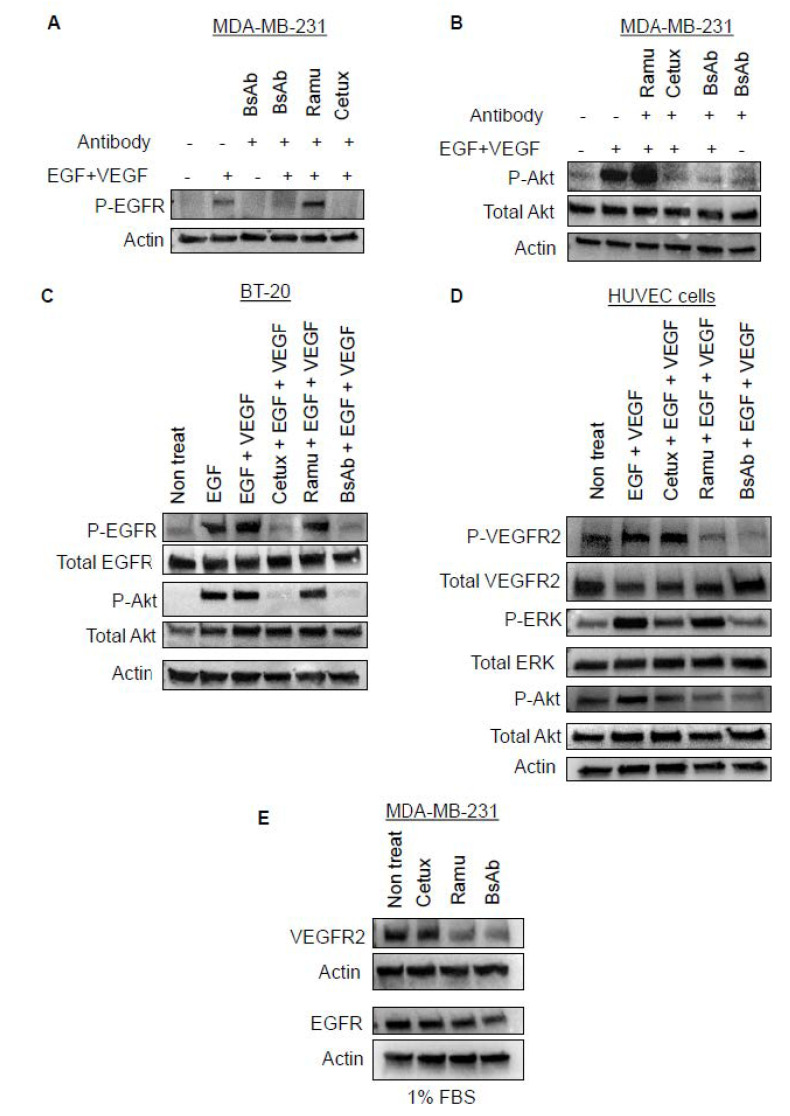
Anti-EGFR/VEGFR2 BsAb inhibited ligand-induced activation of EGFR and VEGFR2. (**A**) MDA-MB-231 cells were serum starved overnight and then pre-treated with different antibodies at concentration of 10 µg/mL for 1 h followed by a co-treatment of EGF and VEGF-A (EGF + VEGF) at a concentration of 50 ng/mL each for 30 min. After treatments, WCL was prepared and subjected to Western blot analysis to determine the phosphorylation of EGFR. (**B**) MDA-MB-231 cells were treated as described in A and then phosphorylation levels of Akt was determined by western blot analysis. (**C**) BT-20 cells were exposed to the treatment conditions as described in the A and analyzed by western blotting to assess the phosphorylated and total levels of EGFR, ERK and AKT. (**D**) HUVEC cells were treated with antibodies and EGF + VEGF as described in A. After treatments, WCL was analyzed by western blot to determine the phosphorylated and total levels of VEGFR2, ERK, Akt using respective antibodies. (**E**) MDA-MB-231 cells were serum starved overnight in media containing 1% FBS and then treated with cetuximab, ramucirumab, anti-EGFR/VEGFR2 or left untreated. Western blotting was carried out to evaluate the expression of total VEGFR2. Unprocessed western blot images and available in Figures S5 and S6.

To explore the mechanisms of anti-tumor activities of the anti-EGFR/VEGFR2 BsAb, EGFR and VEGFR2 phosphorylation and their downstream signaling molecules were examined. Treatments with combination of ligands (EGF + VEGF) dramatically enhanced the phosphorylation levels of EGFR at Y1086 phosphorylation site in both MDA-MB-231 and BT-20 cells (Figure S3A,B). As shown in Figure 5A and Figure S5, anti-EGFR/VEGFR2 BsAb blocked the EGF + VEGF induced-phosphorylation of EGFR in MDA-MB-231 cells which was similar to cetuximab-mediated effect. The effect of anti-EGFR/VEGFR2 BsAb on Akt, a major downstream signal transduction molecule associated with EGFR signaling, was also determined. As shown in Figure 5B and Figure S5, the Akt signaling was dramatically activated by EGF + VEGF stimulation, which was effectively blocked by anti-EGFR/VEGFR2 BsAb and cetuximab treatment but not with ramucirumab treatment in MDA-MB-231 cells. Similar effect of anti-EGFR/VEGFR2 BsAb treatment on EGFR and Akt phosphorylation was also observed in BT-20 cells (Figure 5C and Figure S6). Furthermore, we studied the effect of anti-EGFR/VEGFR2 BsAb on VEGFR2 signaling in HUVEC cells after stimulation with EGF + VEGF. The treatment with EGF + VEGF considerably enhanced the phosphorylation levels of VEGFR2 in HUVEC cells (Figure 5D and Figure S6). The upregulated VEGFR2 phosphorylation was greatly diminished in anti-EGFR/VEGFR2 BsAb-treated HUVEC cells and moderately reduced in ramucirumab-treated cells. Furthermore, the phosphorylation of downstream signaling molecules, ERK and AKT which was enhanced by ligand stimulation, was effectively inhibited by anti-EGFR/VEGFR2 BsAb in HUVEC cells (Figure 5D). However, ramucirumab had no inhibitory effect on ligands-induced phosphorylation of ERK, but downregulated AKT activity, whereas cetuximab inhibited ERK phosphorylation induced by ligands, but had a weaker inhibitory effect on AKT phosphorylation (Figure 5D). These data suggest that anti-EGFR/VEGFR2 BsAb downregulated VEGFR2 signaling in HUVEC cells and exhibited a better inhibitory activity of downstream signaling pathways including ERK and AKT. We also examined whether anti-EGFR/VEGFR2 BsAb or parental antibody have any effects on the protein levels of EGFR and VEGFR2 in MDA-MB-231 cells. As shown in Figure 5E and Figure S7, both ramucirumab and anti-EGFR/VEGFR2 BsAb dramatically downregulated the protein levels of VEGFR2 in MDA-MB-231 cells (Figure 5E). Anti-EGFR/VEGFR2 BsAb also reduced the protein levels of EGFR. In contrast, cetuximab had no effects on the protein levels of EGFR in MDA-MB-231 cells (Figure 5E). Findings shown in Figure 5E support animal data shown in Figure 4A, such that anti-EGFR/VEGFR2 BsAb demonstrated more potent inhibitory activity than corresponding mAbs toward tumor grow. This synergistic activity exhibited by anti-EGFR/VEGFR2 is most likely via its direct inhibition of EGFR and VEGFR2 signaling pathways of tumor cells or block autocrine mechanism since MDA-MD-231 cells secret VEGF and ramucirumab does not bind to mouse VEGFR2 to interfere angiogenic activity toward tumors. Taken together, these data demonstrate that cetuximab-arm of anti-EGFR/VEGFR2 BsAb function effectively by blocking EGFR-mediated signaling in cancer cells and ramucirumab-scFv segment of anti-EGFR/VEGFR2 BsAb can act convincingly by dysregulating VEGFR2-mediated activity in both MDA-MD-231 and HUVEC cells. These coordinated multifunctional mechanisms mediated by anti-EGFR/VEGFR2 were not achieved by its corresponding mAbs.

### 2.6. Anti-EGFR/VEGFR2 BsAb Impairs Paracrine VEGFR2 Signaling in HUVEC Cells Induced by Cancer Cell Media

**Figure 6:**
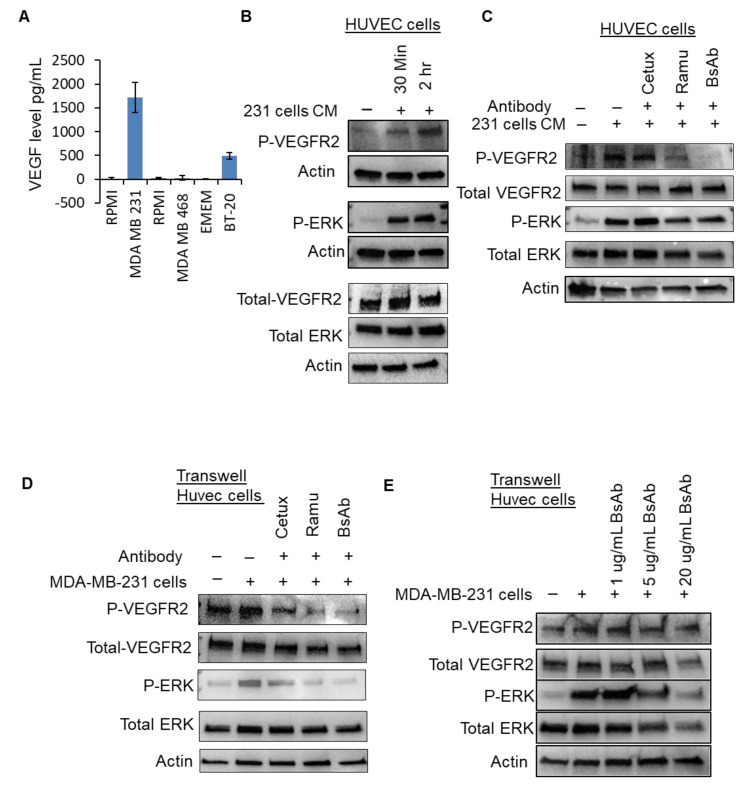
Anti-EGFR/VEGFR2 BsAb downregulates cancer cell-induced VEGFR2 signaling in HUVEC cells (**A**) The levels of VEGF-A were determined by ELISA assay in supernatants of MDA-MB-231, MDA-MB-468 and BT-20 cells after culturing the cells in a 6-well-plate for 2 days at 37 °C. (**B**) MDA-MB-231 cells were cultured for 2 days in serum free media and then conditioned media was harvested. HUVEC cells were incubated for 30 min and 2 h with the conditioned media obtained from MDA-MB-231. After incubation, Western blot analysis was performed to assess the phosphorylated and total levels of VEGFR2 and ERK. (**C**) HUVEC cells were pretreated with cetuximab, ramucirumab, anti-EGFR/VEGFR2 BsAb or left untreated and then incubated with conditioned media obtained from MDA-MB-231 cells. Post-incubation, western blotting was performed to assess the phosphorylated and total levels of VEGFR2 and ERK. (**D**) HUVEC cells were cultured in the top chamber of trans-well co-culture plates and MDAMB231 cells were cultured in a separate 6-well plate in serum free media overnight. Both HUVEC and MDAMB231 cells were then treated with cetuximab, ramucirumab, anti-EGFR/VEGFR2 BsAb for 1 h or left untreated. After treatments, the trans-well upper chamber inserts containing HUVEC were placed on top of 6-well plate containing MDA-MB-231 cells for 30 min. WCL of HUVEC cells was collected and subjected to Western blot analysis to evaluate the phosphorylated levels of ERK and VEGFR2. (**E**) HUVEC cells and MDA-MB-231 cells were cultured in trans-well co-culture plate as described in D and treated with 1, 5 and 20 µg/mL of anti-EGFR/VEGFR2 BsAb. WCL of HUVEC cells was then subjected to Western blot analysis to assess the levels of ERK and VEGFR2. Unprocessed western blot images are available in Figures S7 and S8.

In the paracrine model, tumor cells produce VEGF-A, which binds with VEGFRs on the stromal, endothelial and tumor cells and promote the neovascularization to support tumor proliferation, growth and metastasis. In our investigation, we sought to determine if VEGF-A secreted from TNBC cells could affect VEGFR2 signaling on HUVEC cells and assessed whether anti-EGFR/VEGFR2 BsAb could modulate this process. For this purpose, we assessed the levels of VEGF-A secreted by TNBC cells and found that MDA-MB-231 cells produced significantly high amount of VEGF-A in media (Figure 6A). Next, we exposed the HUVEC cells to the conditioned media obtained from 48 h-cultured MDA-MB-231 cells and observed that conditioned media from MDA-MB-231 cells can activate VEGFR2 signaling in HUVEC cells (Figure 6B and Figure S7). Furthermore, we tested whether activated VEGFR2 signaling by conditioned media can be modulated by anti-EGFR/VEGFR2 BsAb treatment. Data from Western blotting show that anti-EGFR/VEGFR2 BsAb remarkably downregulated the VEGFR2 phosphorylation in HUVEC cells which was activated by conditioned media obtained from MDA-MB-231 cells (Figure 6C and Figure S7). Ramucirumab moderately inhibited VEGFR2 phosphorylation, whereas cetuximab did not have any effect. Downstream signaling molecule ERK was also considerably downregulated by anti-EGFR/VEGFR2 BsAb treatment in HUVEC cells. Next, we used trans-well non-contacting co-culturing system to investigate the effect of anti-EGFR/VEGFR2 BsAb on VEGFR2 signaling in HUVEC cells. Anti-EGFR/VEGFR2 BsAb substantially downregulated the VEGFR2 and ERK phosphorylation in HUVEC cells in trans-well culturing condition (Figure 6D and Figure S8). Additionally, the effect of anti-EGFR/VEGFR2 BsAb on VEGFR2 and ERK signaling was dose-dependent, as observed in Figure 6E and Figure S8. Taken together, these data indicate that anti-EGFR/VEGFR2 BsAb very efficiently impaired the paracrine effects of VEGF/VEGFR2 signaling to exert its antitumor activities.

## 3. Discussion

BsAbs are protein agents genetically-engineered to target two antigens simultaneously and to mediate multiple biological effects. The dual-targeting capacity may render BsAbs advantageous over conventional monospecific antibodies. Development of single BsAb is far less complex and potentially more cost effective than developing two or more individual mAbs [22]. In this investigation, we have generated a tetravalent anti-EGFR/VEGFR2 BsAb with IgG-like format, which has ability to simultaneously block EGFR and VEGFR2 signaling pathways and demonstrate superior anti-tumor activity in vitro and in vivo models on TNBC. Anti-EGFR/VEGFR2 BsAb mimic the functions of both cetuximab and ramucirumab by blocking ligand-mediated activation of EGFR signaling in TNBC cells and inhibited VEGFR2 signaling in HUVEC cells, suggesting that bispecific functionality of anti-EGFR/VEGFR2 BsAb can eliminate the requirement of combining two mAbs to achieve desired anti-tumor effects.

The rationale to combine dual targeting of EGFR and VEGFR2 derived from the studies which demonstrate that there is significant cross-talk between these two signaling cascades to mediated tumor growth, survival and angiogenesis. Both EGFR and VEGFR2 contribute towards the activation of ERK and/or PI3 K/Akt signaling [23,24]. Both EGFR and VEGFR2 regulate downstream PI3 K/AKT and MAPK signaling pathways in cancer cells that co-express both EGFR and VEGFR2 [25,26,27]. Blocking either EGFR or VEGFR2 alone may still allow PI3 K/AKT and/or MAPK to remain activated, hence tumor growth may not be efficiently inhibited by single therapy. Additionally, in-vivo studies showed that EGFR-directed TKIs may induce a switch from EGFR dependence to VEGFR signaling in tumor-associated endothelial cells which further shows the ineffectiveness of single-agent therapy [28]. Given these strong justifications to dual target EGFR and VEGFR2, we uniquely designed anti-EGFR/VEGFR2 BsAb which is capable of simultaneous blocking of EGF/EGFR and VEGF/VEGFR2 signaling, thus providing more comprehensive suppression of two oncogenic signaling pathways for antitumor effects.

The one arm of anti-EGFR/VEGFR2 BsAb binds to EGFR in a manner similar to cetuximab and mimics cetuximab-mediated functions. Cetuximab, which is FDA-approved mAb to treat a variety of human cancers, binds to the extracellular domain of EGFR, and mediates its anti-tumor properties by apoptosis induction, cell-cycle arrest, metastasis inhibition, antibody and complement-mediated cytotoxicity [29,30]. Despite the promising results in pre-clinical settings, published reports indicate that cetuximab therapy did not yield favorable outcome in large clinical trials of metastatic EGFR expressing breast cancers [12]. The lack of response to EGFR-targeted therapy could be attributed to the fact that TNBCs, which commonly overexpress EGFR, are not entirely dependent on EGFR signaling for their survival [9]. Resistance to cetuximab therapy has been extensively studied in colorectal cancers, which shows that amplification of MET or HER2, deletion of PTEN, activation of KRAS, PIK3 CA and other oncogenes could cause persistent activation of growth signaling pathways in cancer cells, thus anti-EGFR mAbs therapy remains ineffective for such tumors [31]. The anti-EGFR/VEGFR2 BsAb described here demonstrates anti-tumor activity in TNBC cells including MDA-MB-231 cells which harbor KRAS mutation [32]. Our data show that anti-EGFR/VEGFR2 BsAb function similarly or better than cetuximab in blocking ligand-mediated phosphorylation/activation of EGFR signaling and its downstream molecules ERK and Akt in TNBC cells.

The second arm of anti-EGFR/VEGFR2 BsAb binds to VEGFR2 and mimics ramucirumab function in downregulating VEGFR2-mediated signaling. TNBC cells express VEGFR2 and secrete VEGF that not only activates endothelial cells in paracrine manner, which express high levels of VEGFR2, but also promotes cellular growth of TNBC cells expressing VEGFR2 in autocrine fashion. Our data show that TNBC cells (MDA-MB-231 and BT-20 cells) secrete significant amount of VEGF in cell culture media. When HUVEC cells were exposed to conditioned media obtained from MDA-MB-231 cells or cultured in trans-well coculture system, VEGFR2 signaling in HUVEC cells was activated via paracrine mechanism. This paracrine activation of VEGFR2 signaling was downregulated by anti-EGFR/VEGFR2 BsAb in a similar fashion to ramucirumab.

Collectively, our novel findings from in-vitro experiments demonstrate that anti-EGFR/VEGFR2 BsAb executes its growth inhibitory bispecific effects via not only direct inhibition of both EGFR and VEGFR2 signaling in TNBC cells, but also disruption of autocrine mechanism in TNBC cells and paracrine pathways in endothelial cells, both of which are mediated by VEGF/VEGFR2.

Our in-vivo data shows that anti-EGFR/VEGFR2 BsAb had a better inhibitory activity in controlling the growth of tumor xenografts bearing MDA-MB-231 cells in comparison to single targeting mAb treatment alone. Pre-clinical study for ramucirumab utilized a rat-mouse VEGFR2 specific mAb, DC101, because ramucirumab does not cross-react to mouse VEGFR2 [33,34]. In vivo experiments utilizing DC101 as VEGFR2 blocker investigated the inhibition of angiogenesis and decreased endothelial cell survival in a murine model of colon carcinoma liver metastases [34]. In our xenograft study, we used human ramucirumab, therefore it is unlikely that ramucirumab arm of anti-EGFR/VEGFR2 BsAb mediated angiogenesis inhibition to suppress the growth of tumor xenografts in our study. Instead, we speculate that anti-EGFR/VEGFR2 BsAb exerts its anti-tumor activity in our in-vivo mouse study via inhibition of EGFR signaling and disruption of autocrine and/or paracrine mechanisms modulated by VEGF/VEGFR2, which further supports our findings from cellular models.

## 4. Materials and Methods

### 4.1. Cell Culture and Reagents

The TNBC cell lines, MDA-MB-231, BT-20, MDA-MB-468, BT549 and HS578 T, were purchased from American Type Culture Collection (ATCC, Manassas, VA, USA) and maintained in culture media and supplements as described previously [7]. The adherent human umbilical vein endothelial cells (HUVEC) were also purchased from ATCC and propagated in endothelial culture media supplemented with growth factors and heparin purchased from Corning (Glendale, AZ, USA). Expi293 F cells were purchased from ThermoFisher Scientific (Waltham, MA, USA) and maintained in the suspension culture using Expi293 Expression Medium (ThermoFisher Scientific, Waltham, MA, USA). Therapeutic monoclonal antibodies cetuximab (Erbitux^®^) and ramucirumab (Cyramza^®^) were purchased from the FDA-designated pharmacy (McKesson, Irving, TX, USA). The EGF and VEGF-A was obtained from RayBiotech (Peachtree Corners, GA, USA).

### 4.2. Construction, Expression and Purification of the Anti-EGFR/VEGFR2 BsAb

The full-length IgG sequence of cetuximab and single-chain variable fragment (scFv) sequence of ramucirumab were derived from publicly available International Immunogenetics Information System (http://www.imgt.org) database. The scFv genes of ramucirumab was designed as V_H_–V_L_ orientation, with a (Gly_4_ Ser)_4_ linker in between. The genes were chemically synthesized by GeneArt Gene Synthesis at Thermo Fisher Scientific and tethered to the n-terminal of cetuximab H-chain with a (Gly_3_ Ser)_4_ linker for BsAb by overlapping PCR. The H-chains were then cloned into pCDNA3.1 (+) between HindIII site and XhoI site, and sequenced (Biotechnology Resources (FBR) Core facility, Center for Biologics Evaluation and Research (CBER, FDA, Silver Spring, MD, USA). The transfection and purification of anti-EGFR/VEGFR2 BsAb was performed as described before [35]. The two plasmids of anti-EGFR/VEGFR2 BsAb encoding heavy- and light-chain were transiently co-transfected into Expi293 cells using Expifectamine 293 Transfection Kit (Thermo Fisher Scientific, Rockford, IL, USA) as per manufacturer’s instructions. The Expi293 F supernatants were harvested on day 6 post-transfection, centrifuged at 3200× *g* for 20 min and filtered using 0.22 µm filter unit. The purification of anti-EGFR/VEGFR2 BsAb was performed by affinity chromatography using immobilized protein-A agarose resin (Thermo Fisher Scientific) by passing through disposable plastic gravity flow columns. The bound recombinant anti-EGFR/VEGFR2 BsAb was eluted in low pH glycine-HCl elution buffer (pH 2.7) and neutralized in Tris-HCl solution (pH 7.6). The antibody was concentrated by Amicon^®^ Ultra-15 Centrifugal Filter Unit (EMD Millipore, Burlington, MA, USA) in low endotoxin PBS yielding the final concentration of anti-EGFR/VEGFR2 BsAb of 0.5–1 mg/mL.

### 4.3. ELISA Assay

The standard ELISA assay was performed to detect the binding of mAbs and anti-EGFR/VEGFR2 BsAb to receptors, EGFR or VEGFR2. Briefly, 96-well microtiter plate was coated with 10 µg of EGFR or VEGFR2 for 1 h at 37 °C. The wells were washed and then various concentrations of primary antibodies (cetuximab, ramucirumab or anti-EGFR/VEGFR2 BsAb) were added to the plate. The plate was incubated for 1 h at 37 °C. Then a peroxidase-conjugated goat antihuman immunoglobulin G was applied for 1 h at 37 °C followed by substrate solution for 30 min. Stop solution was added to quench the reaction and then color development was assessed by measuring absorption at 450 nm using a spectrophotometer. In the ELISA assay for VEGF concentration, 5 × 10^5^ cells were cultured in a 6-well-plate at 37 °C with culture media. Then the supernatants were collected and tested for human VEGF expression with a Human VEGF PicoKin ELISA Kit (Boster Bio, Pleasanton, CA, USA) as per manufacturer’s instructions.

### 4.4. Flow Cytometry Binding Assay

The binding of anti-EGFR/VEGFR2 BsAb with cell surface EGFR and VEGFR2 expressing in MDA-MB-231, BT-20, MDA-MB-468 and HUVEC cells was detected using flow cytometry. Briefly, cells were harvested by trypsinization and washed twice with PBS. The staining for live-dead cells was done by incubating cells with 1 µL violet stain for 30 min on ice. Cells were washed twice with FACS buffer (1% FBS in PBS) and then incubated with 1 µg of ramucirumab, cetuximab or anti-EGFR/VEGFR2 BsAb for 30 min on ice. The human IgG was used as isotype control. After primary antibody incubation, cells were washed twice with FACS buffer and incubated with anti-human FITC secondary antibody for 30 min on ice. Cells were washed with FACS buffer and resuspended in 250 µL FACS buffer and analyzed immediately using a LSR Fortessa (BD Bioscience, San Jose, CA, USA) flow cytometer.

### 4.5. Biacore Binding Kinetics Assay

Surface plasmon resonance (SPR) measurements were performed for binding kinetics analyses with a Biacore T200 optical biosensor instrument (GE Healthcare, Piscataway, NJ, USA). Biacore T200 optical biosensor utilizes SPR technology to perform the real-time detection and monitoring of biomolecular binding events. The CM5 sensor chip surface were activated by injection of 35 μL of a solution containing 0.2 M *n*-(3-dimethylaminopropyl)-*n*-ethylcarbodiimide and 0.05 M *n*-hydroxysuccinimide. Next, 20 μg/mL of protein A in 10 mM sodium-acetate buffer (GE Healthcare, Chicago, IL, USA, pH 4.5) could flow over the chip surface until the desired level of response units of reacted protein (>200 Response Unit, RU) was achieved. Various concentrations of recombinant human VEGFR2 and EGFR were diluted in 1× HBST buffer (0.01 HEPES, 0.15 M NaCl, 0.05% Tween) and immobilized on activated CM5 chip surface at for 3 min. Binding kinetics was measured by passing the antibodies (cetuximab, ramucirumab and anti-EGFR/VEGFR2 BsAb) at 8 µg/mL over the chip surface for 3 min. The dissociation of bound analytes was monitored while the surface was washed for 10 min. The CM5 sensor chip surfaces were regenerated by injecting regeneration buffer (glycine, pH 1.5, GE Healthcare). The kinetic data was analyzed with BiacoreT200 evaluation software version 3.0 and were fitted to the 1:1 Langmuir binding model. K_a_ and K_d_ are kinetic rate constants where K_a_ denotes association rate constant and K_d_ denotes dissociation rate constants. Equilibrium dissociation constant (K_d_), which is a measurement of binding affinity, is the ratio of rate constants (K_d_/K_a_) [36].

### 4.6. Western Blot Analysis

Cell lysates were prepared in NP-40 cell lysis buffer and subjected to 4–15% gradient SDS-PAGE gel separation as described previously [37]. After separation, gels were transferred to a 0.22-μm PVDF membrane using Trans-Blot Turbo Transfer System (BioRad, Hercules, CA, USA) and blocked with 5% non-fat milk in TBST for 1 h at room temperature. The membranes were incubated with primary antibodies overnight at 4 °C and HRP-conjugated secondary antibodies for 1 h at room temperature. Protein bands were visualized by ChemiDoc MP gel imaging system (BioRad) after incubating with chemiluminescence substrate. The primary antibodies directed against EGFR, phospho-EGFR, VEGFR2, phospho-VEGFR2, ERK, phospho-ERK, Akt and Phospho-Akt were purchased from Cell Signaling Technologies. The actin antibody and HRP-conjugated secondary antibodies were purchased from Sigma-Aldrich (St. Louis, MO, USA).

### 4.7. Tumor Xenograft Model

All animal experiments were performed in accordance with animal protocol #WO-2018–26, which was approved by the United States Food and Drug Administration (FDA) Institutional Animal Care and Use Committee, in accordance with the U.S. Public Health Service Policy on Humane Care and Use of Laboratory Animals and described previously [38]. Four to six weeks old female athymic nude mice (Jacksons Lab, Bar Harbor, ME, USA) were given subcutaneous injections of MDA-MB-231 cells (5 × 10^6^ cells) on the left and right flanks. After 1–2 weeks when the tumor volume reached approximately 50–100 mm^3^, all the tumor-bearing mice were randomly distributed into 4 groups (*n*  =  5/group) and treated for 4 weeks. Group 1 received saline, group 2 received ramucirumab (10 mg/kg), group 3 received cetuximab (10 mg/kg) and group 4 received anti-EGFR/VEGFR2 BsAb (10 mg/kg). Mice were intraperitoneally injected with the drugs twice a week for 4 weeks and tumor volume was measured using digital calipers twice a week post-injection. Tumor volume was calculated as:

length × (width)^2^ × 0.5

where length was the longest distance and width was shortest distance [39].

The body weight of mice was measured twice a week to monitor systemic toxicity. At the end of the treatment course on day 21, tumors were excised from mice and fixed in 10% neutral buffered formalin (Sigma).

### 4.8. Statistical Analysis

The statistical significance was determined by two-tailed student’s *t* test using Microsoft Excel version 1902 and GraphPad Prism version 5.0 (GraphPad Software). The data was presented in the form of mean ± standard deviation and error bars represents standard deviation. The differences between two groups were considered statistically significant when *p* < 0.05 (*, *p* < 0.05; **, *p* < 0.01).

## 5. Conclusions

In summary, we have generated a novel anti-EGFR/VEGFR2 BsAb by fusing single-chain variable fragment from ramucirumab to the heavy chain of cetuximab using a peptide linker. This BsAb binds to both EGFR and VEGFR2 with binding affinity similar to corresponding monoclonal antibodies, cetuximab and ramucirumab. Anti-EGFR/VEGFR2 BsAb showed enhanced anti-tumor activities via multiple mechanisms of action in both in vitro and in vivo models of TNBC, suggesting that co-targeting of EGFR and VEGFR2 with this BsAb can result in synergistic anti-tumor activities than single agent treatment. Anti-EGFR/VEGFR2 BsAb blocks EGF-mediated phosphorylation of EGFR and downstream pathways in TNBC cells and VEGF/VEGFR2-mediated signaling in endothelial cells. Anti-EGFR/VEGFR2 BsAb exhibits bispecific inhibitory effects on TNBC cells via the downregulation of VEGFR2 and an autocrine pathway and on endothelial cells through a paracrine fashion. Given these novel findings, BsAb therapies such as this could be an attractive approach as a targeted therapy to treat TNBC.

## Figures and Tables

**Table 1:** Median fluorescence intensity as detected by flow cytometry for binding of anti-EGFR/VEGFR2 BsAb, cetuximab or ramucirumab on the cell surface of MDA-MB-231, BT-20, MDA-MB-468 or HUVEC cells.

Treatments	MDA MB 231	BT-20	MDA-MB-468	HUVEC
Isotype control	10	15.9	10.8	12.4
Cetuximab	670	958	3186	12.4
Ramucirumab	9.86	11.2	14.7	670
Anti-EGFR/VEGFR2 BsAb	725	1054	2930	769

## Data Availability

The data presented in this study are available in the article and Supplementary Materials.

## Supplementary Materials

The following are available online at https://www.mdpi.com/2072-6694/13/5/1027/s1, Figure S1: (A) SPR sensor grams showing the binding kinetics of cetuximab to EGFR using Biacore binding assay, (B) SPR sensor grams showing the binding kinetics of ramucirumab to VEGFR2 using Biacore binding assay, Figure S2: (A) Inhibition of cell proliferation profile of MDA-MB-231 as determined by trypan blue exclusion assay. (B) The inhibition of cell proliferation profile of MDA-MB-468 cells as determined by trypan blue exclusion assay, Figure S3: Activation of EGFR signaling in MDA-MB-231 cells (A) and in BT-20 cells (B) after treatment with EGF, VEGF-A or combination of both (EGF + VEGF), Figure S4: Unprocessed western blot images for Figure 2D and Figure 3A,B, Figure S5: Unprocessed western blot images for Figure 5A,B, Figure S6: Unprocessed western blot images for Figure 5C,D, Figure S7: Unprocessed western blot images for Figure 5E and Figure 6B,C, Figure S8: Unprocessed Western blot images for Figure 6D,E.
