# Supplementary material for: A Novel Bispecific Antibody Targeting EGFR and VEGFR2 Is Effective against Triple Negative Breast Cancer via Multiple Mechanisms of Action
Source: Cancers (Basel). 2021 Mar 1;13(5):1027. doi: 10.3390/cancers13051027 (PMC7957537; doi:10.3390/cancers13051027)
Supplement: Supplementary file 1 [file cancers-13-01027-s001.pdf]

cancers

Supplementary Material: A Novel Bispecific Antibody Targeting EGFR and VEGFR2 Is Effective Against Triple Negative Breast Cancer Via Multiple Mechanisms of Action

Nishant Mohan, Xiao Luo, Yi Shen, Zachary Olson, Atul Agrawal, Yukinori Endo, David S. Rotstein, Lorraine C. Pelosof and Wen Jin Wu

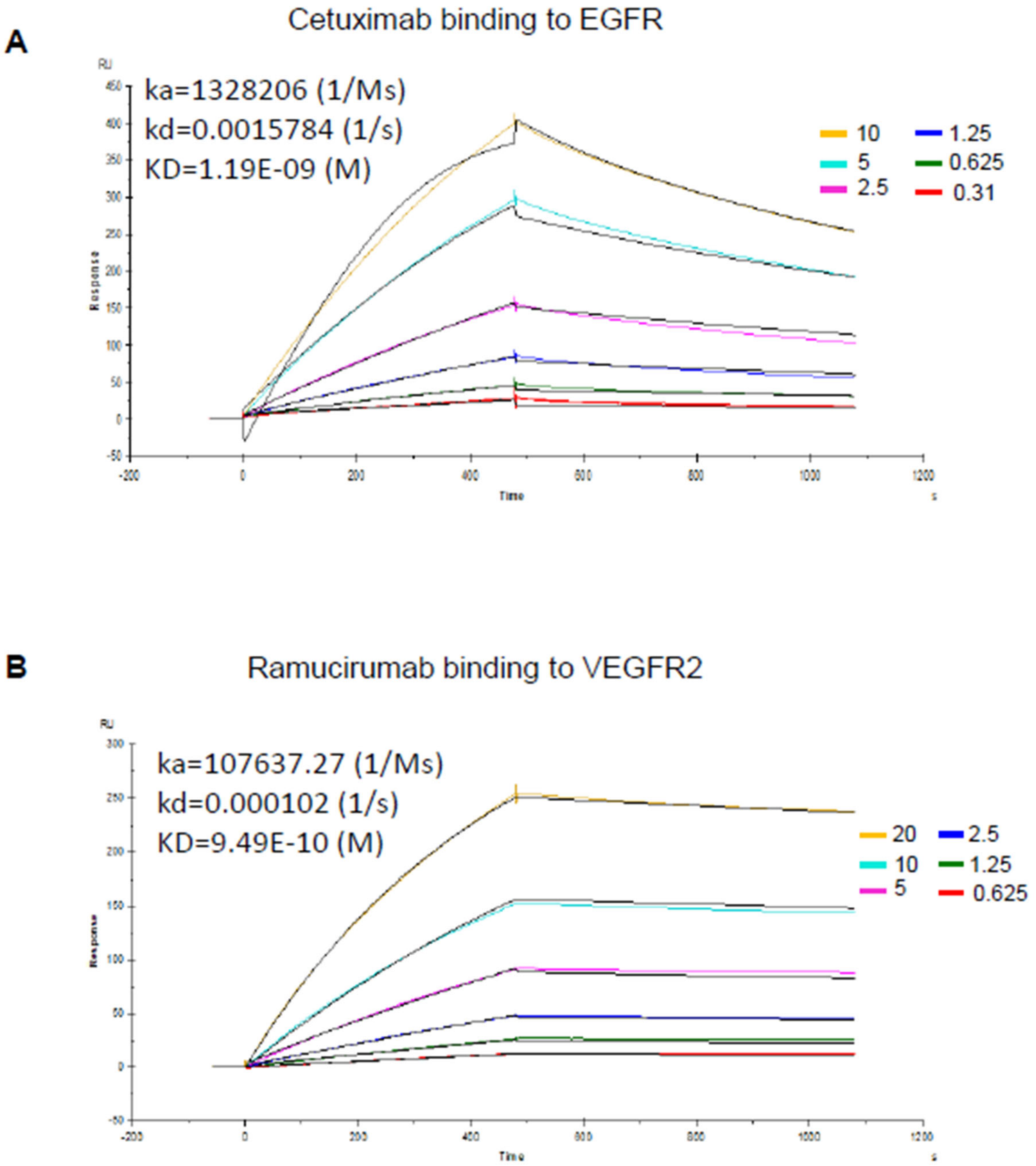

Figure S1. (**A**) SPR sensor grams showing the binding kinetics of cetuximab to EGFR using Biacore binding assay (**B**) SPR sensor grams showing the binding kinetics of ramucirumab to VEGFR2 using Biacore binding assay. Black line represents

the constant concentration of cetuximab or ramucirumab (8µg/ml) and colored lines represent the nM concentrations of antigens (EGFR or VEGFR2) as indicated.

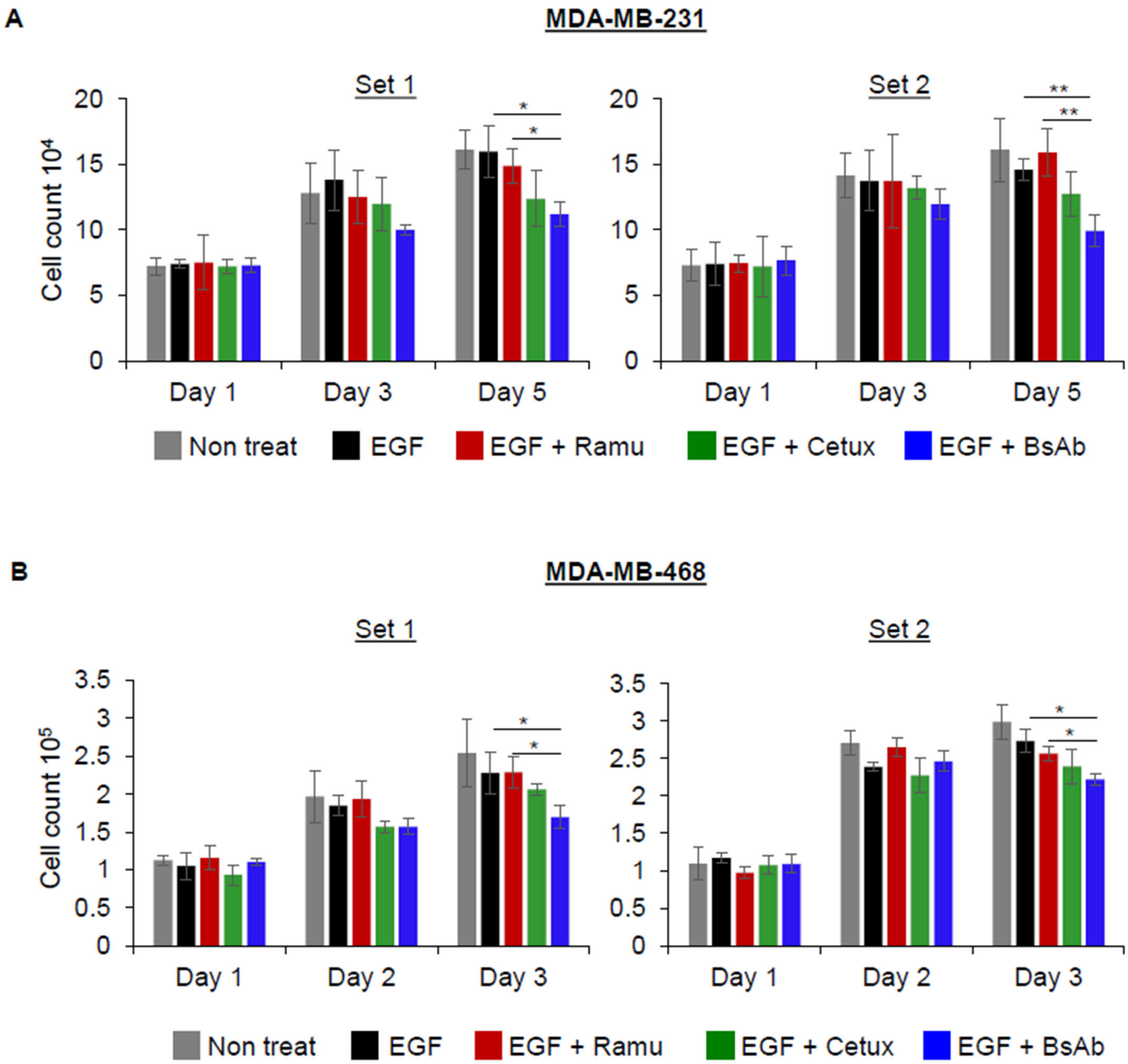

Figure S2. (**A**) Inhibition of cell proliferation profile of MDA-MB-231 as determined by trypan blue exclusion assay. Cells were seeded in 24-well plates in media containing 10% FBS. Next day, cells were pre-treated with ramucirumab, cetuximab or anti-EGFR/VEGFR2 BsAb at 10µg/ml concentration for 1 hour, and then EGF was added into wells at 50 ng/ml. Cell culture media were then changed every other day with fresh media containing antibodies and/or EGF. Cell counting was performed on day 1, 3 and 5 after treatment. Non-treat control cells did not receive any treatment except that the cell culture media were changed every other day. Data were from two independent experiments in triplicates. (**B**) The inhibition of cell proliferation profile of MDA-MB-468 cells as determined by trypan blue exclusion assay. The experimental procedure was essentially the same as that described in (**A**) except that cell counting was performed on day 1, 2 and 3 after treatment. Data were obtained from two independent experiments in triplicates. * $p < 0.05$; ** $p < 0.01$. Uncropped Western Blots are available in Figure S9.

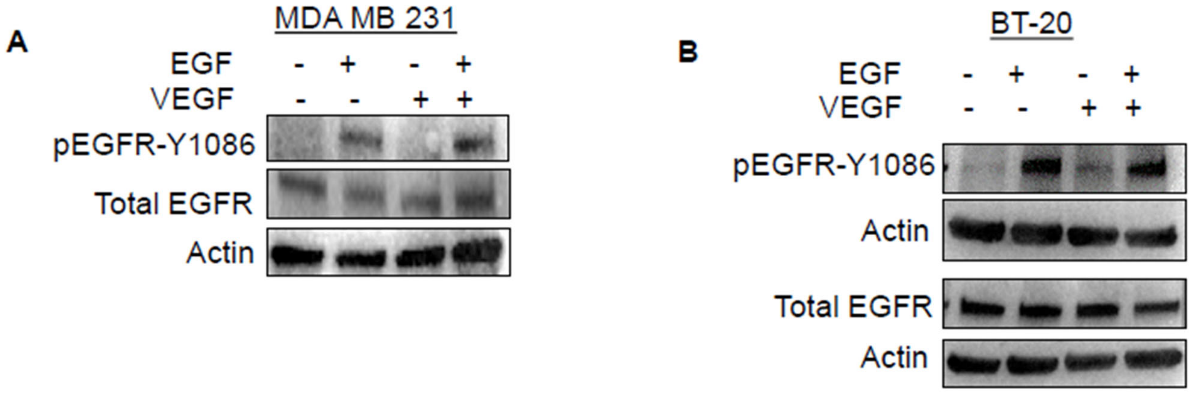

Figure S3. Activation of EGFR signaling in MDA-MB-231 cells (**A**) and in BT-20 cells (**B**) after treatment with EGF, VEGF-A or combination of both (EGF + VEGF). After treatments, WCL was collected and subjected to Western blot analysis to determine the phosphorylated and total levels of EGFR.

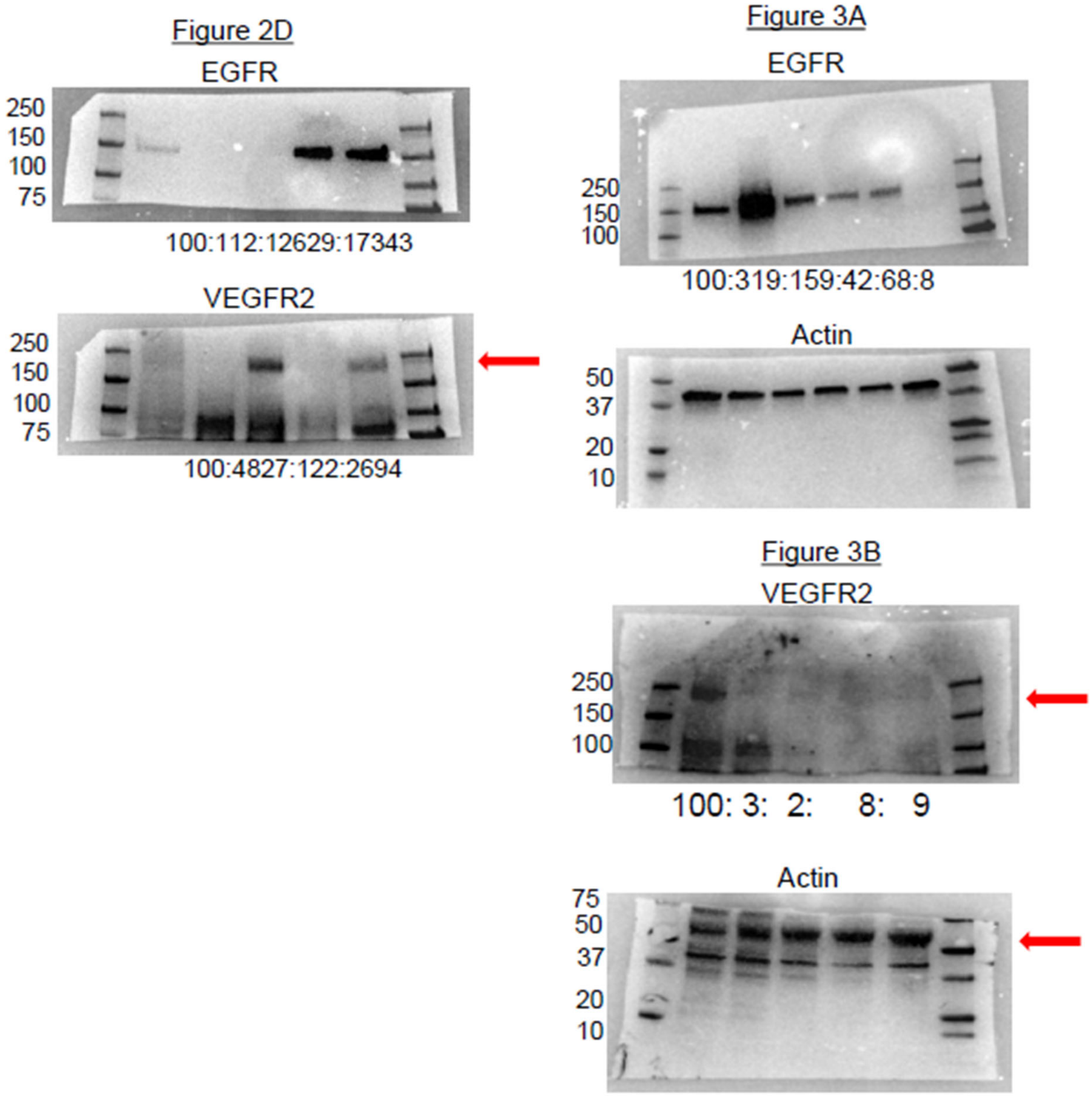

Figure S4. Unprocessed western blot images for Figure 2D, 3A and 3B.

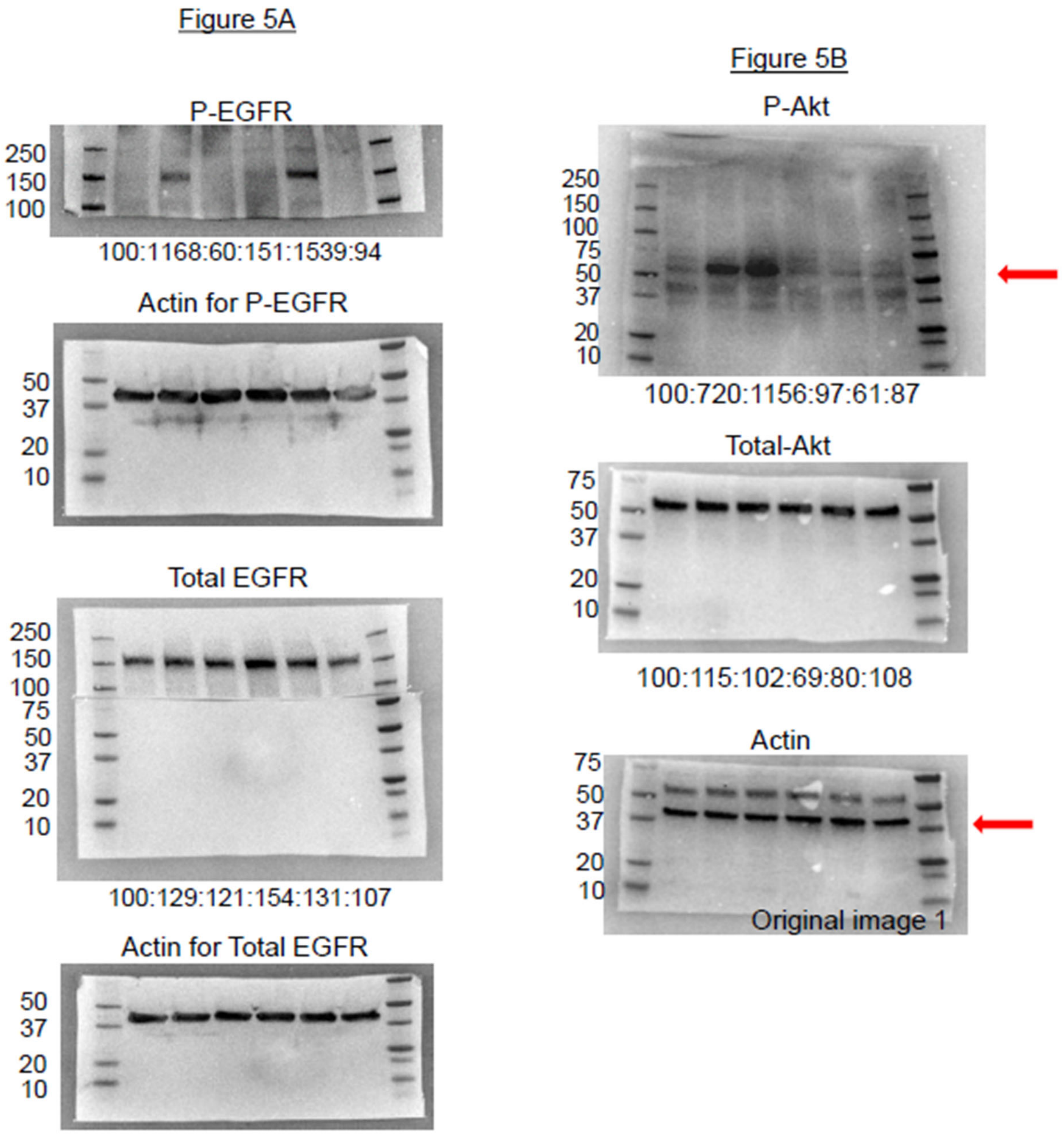

Figure S5. Unprocessed western blot images for Figure 5A and 5B.

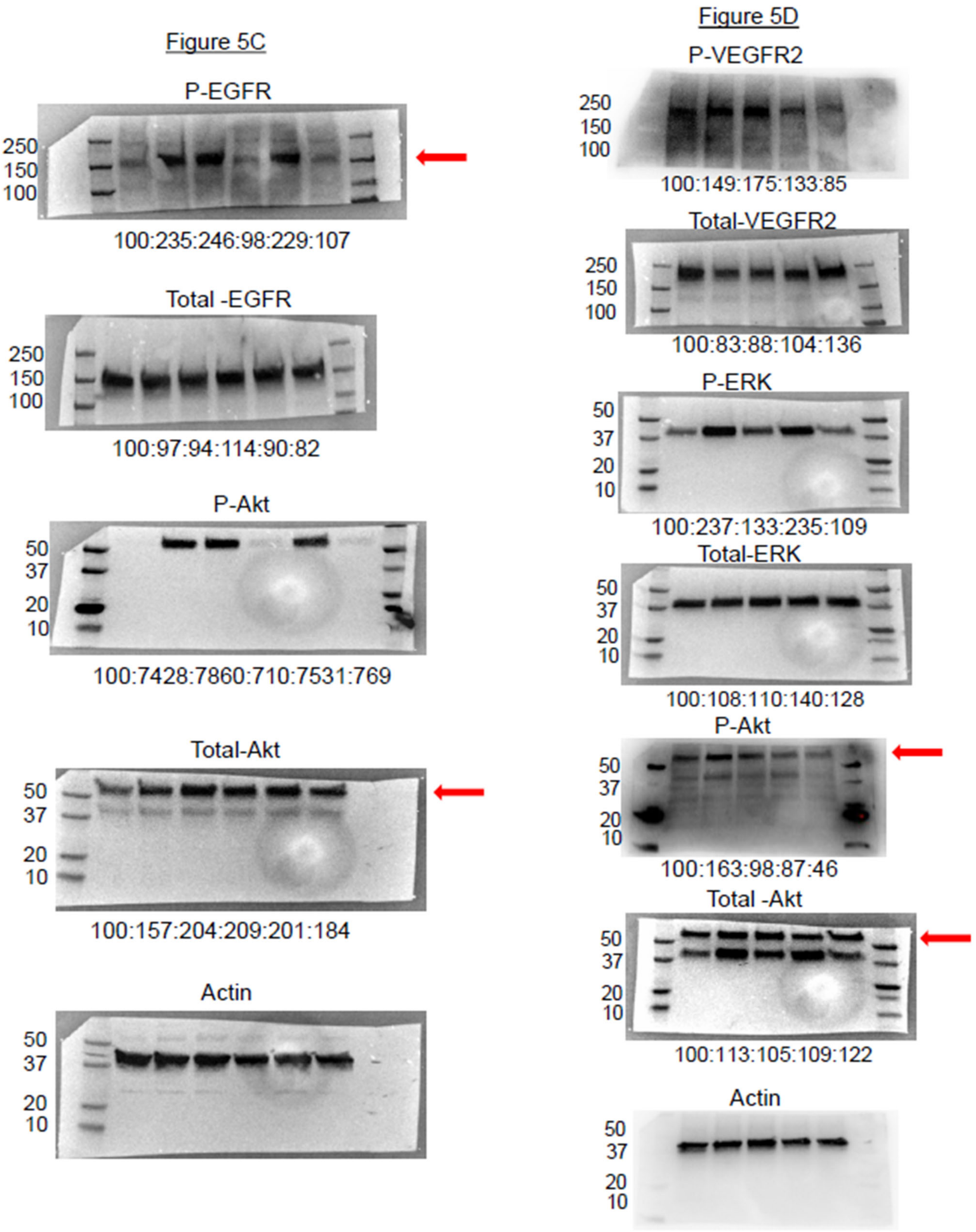

Figure S6. Unprocessed western blot images for Figure 5C and 5D.

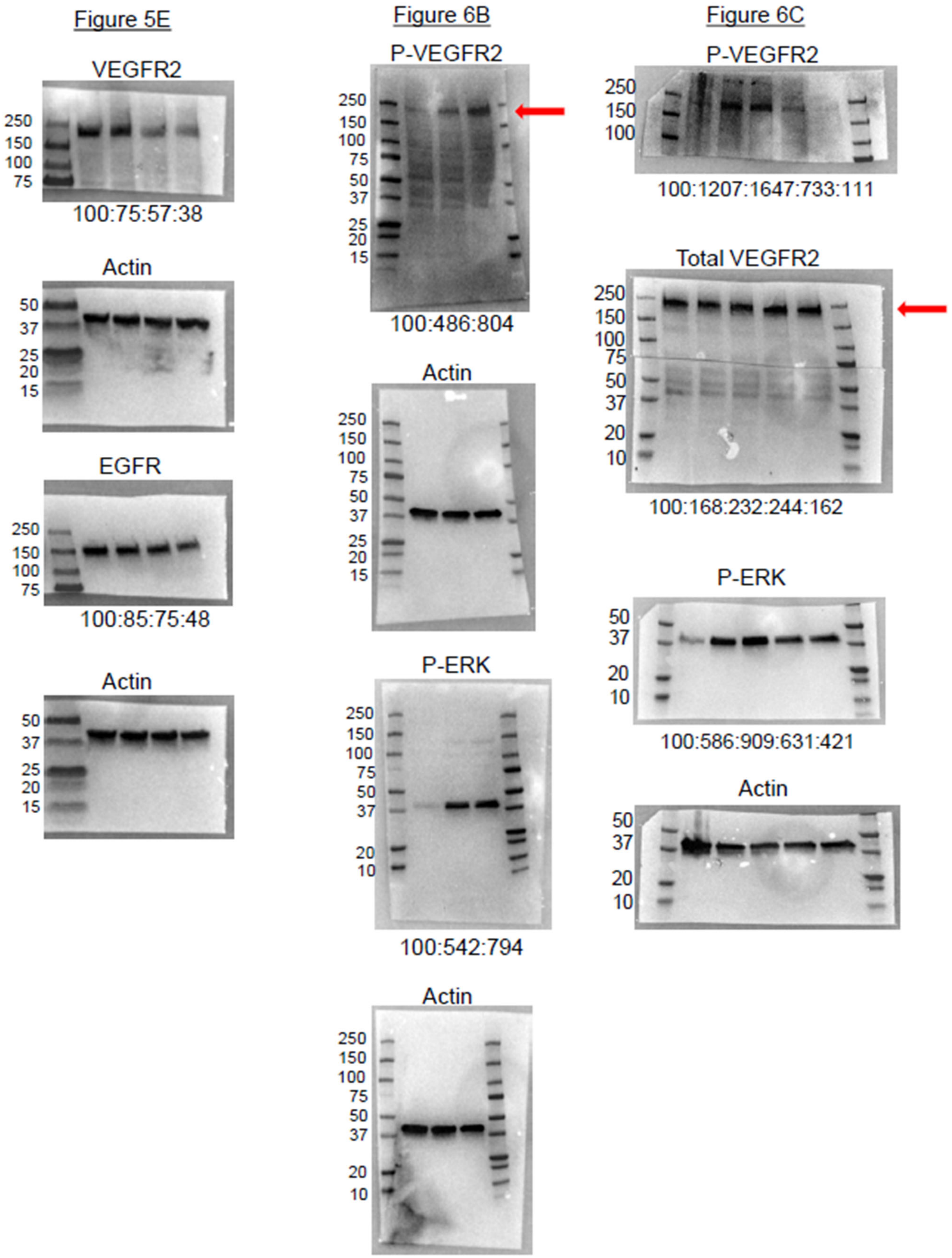

Figure S7. Unprocessed western blot images for Figure 5E, 6B and 6C.

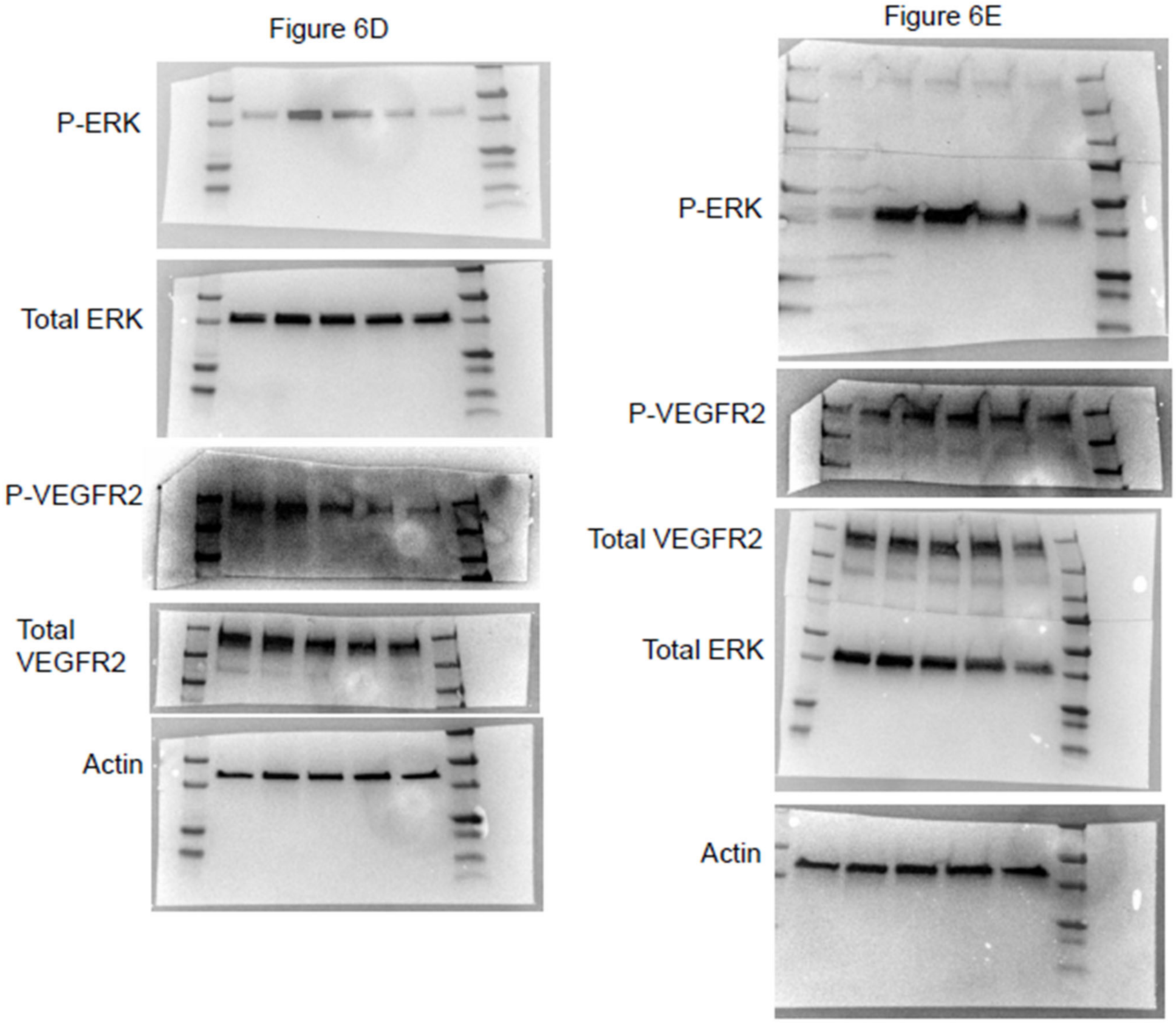

Figure S8. Unprocessed Western blot images for Figure 6D and 6E.

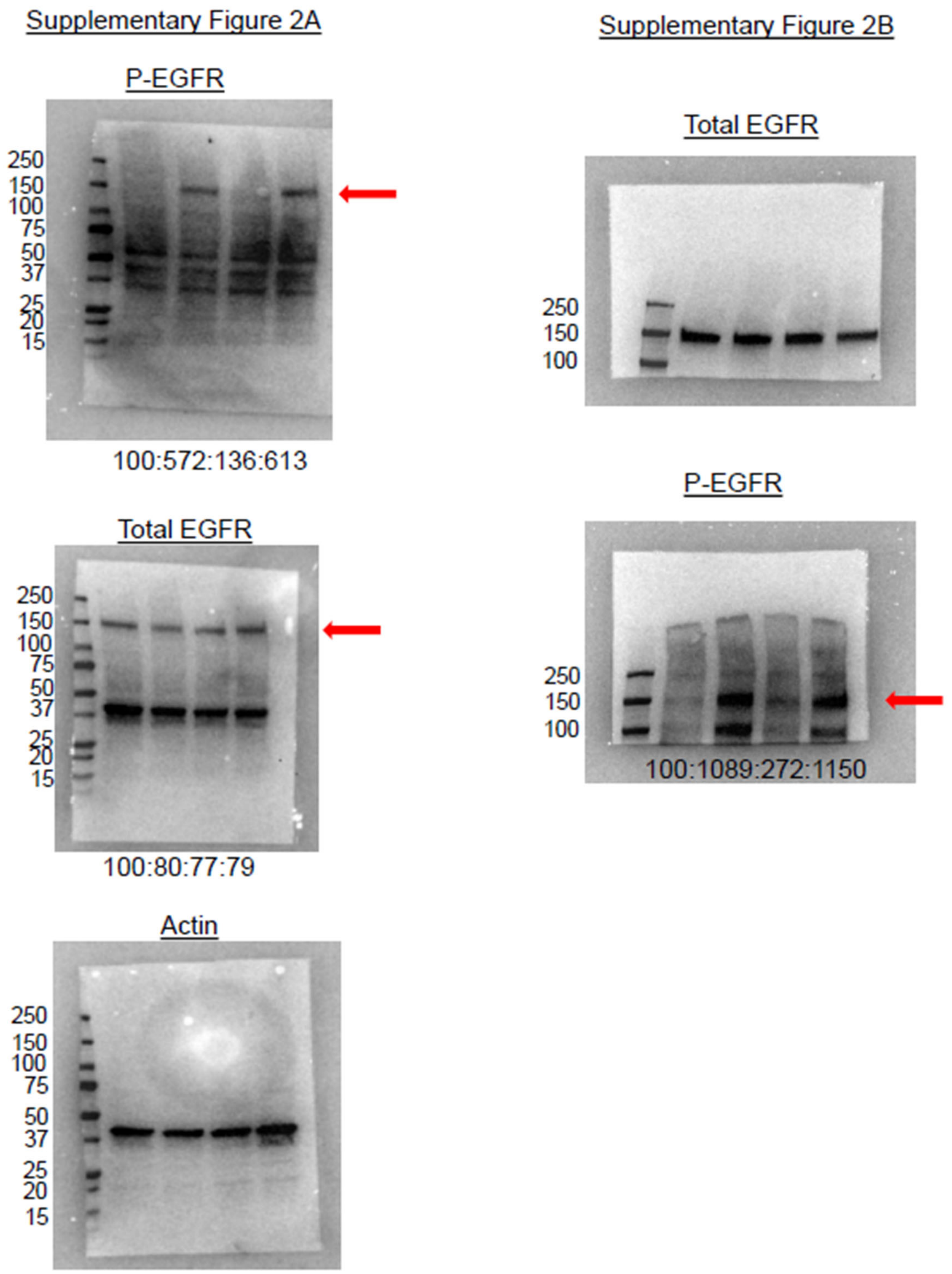

Figure S9. Unprocessed Western blot images for Figure S2A,B.
